# New Method for the Radical Cure of Hydrocele

**Published:** 1844-03-09

**Authors:** 


NEW METHOD FOR THE RADICAL CURE OF HYDROCELE.

At the seance of the Academie de Medicine, 28th November, a letter was read from M. Guillon, on a new method for the radical cure of hydrocele. It consists in introducing into the tunica vaginalis a very small flexible bougie long enough to form several spiral folds in the cavity; and keeping it there till pain and inflammation come on.

The following is the manner in which M. Guillon, performs the operation. He plunges a trocar into the bottom of the tumor; and when about half the liquid has escaped, he introduces the bougie through the canula into the sac, and causes it to form two or three spiral folds in its interior, so as to distend it greatly. He generally lets it remain about eighteen hours, or at least till pain and swelling come on.

*Prov. Med. and Surg. Journ.*

